# Reverse Electrodialysis: Co- and Counterflow Optimization of Multistage Configurations for Maximum Energy Efficiency

**DOI:** 10.3390/membranes10090206

**Published:** 2020-08-28

**Authors:** Joost Veerman

**Affiliations:** RED stack bv, Graaf Adolfstraat 35-G, 8606 BT Sneek, The Netherlands; j.veerman@redstack.nl

**Keywords:** salinity gradient power, salinity gradient energy, renewable energy, blue energy, electrode segmentation, cation exchange membrane, anion exchange membrane

## Abstract

Reverse electrodialysis (RED) is one of the techniques able to harvest energy from the salinity gradient between different salt solutions. There is a tradeoff between efficiency and generated power in a RED stack. This paper focuses on efficiency. A simple model is presented to calculate the efficiency in a co-flow or counterflow operated stack. Moreover, the efficiency can be improved by applying multistaging; the stacks in such a system can also be interconnected externally in co- and counterflow. The four combinations of internally and externally flow modes are the base of further considerations concerning procedures for optimization of these configurations. Three methods for optimization the energy efficiency in a multistage system are discussed: (A) successively maximizing the power of each individual stage, (B) maximizing the power of the whole system by adjusting the electrical current in all stages simultaneously, and (C) maximizing the power of the whole system by adjusting the same current through each stage. Method C is the most attractive because it only requires one converter (cheaper and easier to control) while the results are hardly inferior to B and much better than A. An alternative to multistaging is electrode segmentation and the advantages and disadvantages of both systems are briefly discussed.

## 1. Introduction

Salinity gradient power (SGP) can be harvested from the reversible mixing of feed waters with different salinity. The largest source of salinity gradients are the rivers and seas [1]. It is a sustainable and clean form of energy, producing only electrical energy and brackish water. The theoretical amount of energy content of mixing one cubic meter river water with a large excess of seawater is 2.5 MJ or 1.7 MJ when mixed with the same volume seawater [2]. The global potential of SGP is estimated to be 2.6 TW [3] which is comparable with the current world electricity consumption of three terawatts [4]. The two most important membrane-based technologies used to harvest this energy are reverse electrodialysis (RED) [5,6] and pressure retarded osmosis (PRO) [7]. In RED there is a direct conversion of the exergy of the feed waters into electrical energy and this technique is the subject of this paper. The terms ‘seawater’ and ‘river water’ are used throughout this paper for the high and low concentration flows.

**Figure 1:**
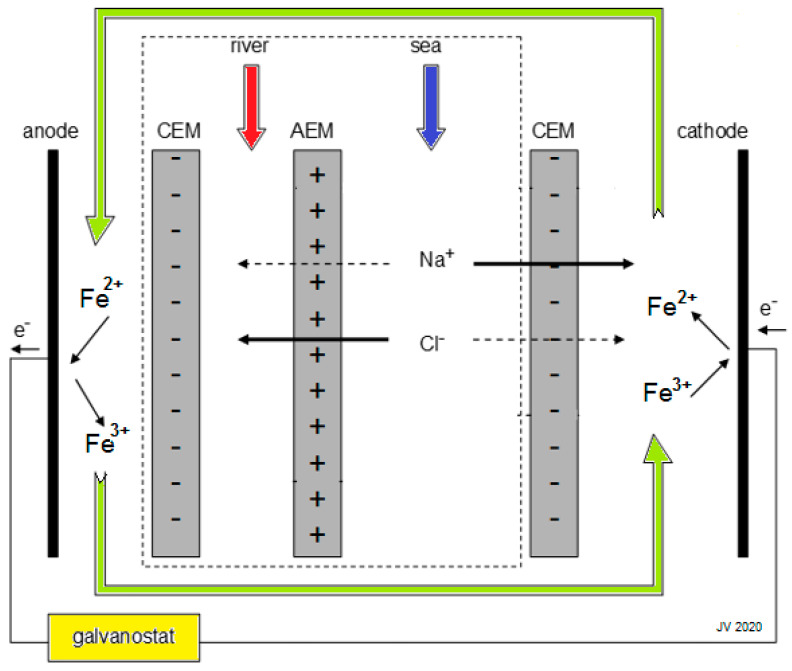
Reverse electrodialysis (RED) stack with only one cell pair (the part within the dashed square). Signs in the membranes represent fixed charges. Wanted movements of ions through the membranes are indicated with solid arrows and unwanted with dashed ones. A solution of a FeCl_2_/FeCl_3_ mixture in a NaCl bulk is used as electrode rinse solution [1].

A RED stack consists of a large number of cell pairs, each containing a cation exchange membrane (CEM), a river water compartment, an anion exchange membrane (EM) and a seawater compartment. The stack is terminated by an electrode compartment with an electrode on each side of the stack (Figure 1). The driving force in the cell is the chemical potential difference between the sea- and river water. Cations diffuse through the CEM from the sea to the river water compartment in one direction and anions through the EM in the opposite direction. The internal ionic current is converted to an external electron current at the electrodes by redox reactions or capacitive mechanisms.

**Figure 2:**
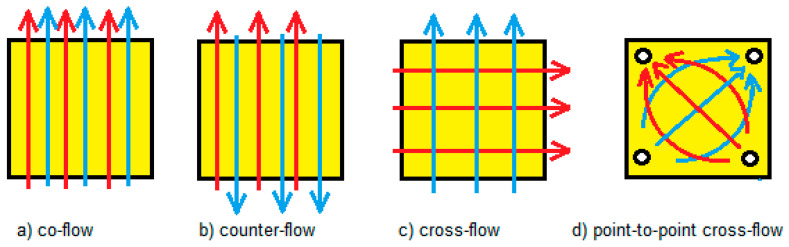
Different flow directions in a RED stack: co-flow (**a**), counterflow (**b**), side-to-side crossflow (**c**), and point-to-point cross-flow (**d**). Red arrows indicate the flow direction in the river water compartments and blue arrows the direction in the seawater compartments.

The high and low concentration flows within a RED can follow different mutual directions. We distinguish co-, cross- and counterflow operation (Figure 2). In addition to the usual (side-to-side) crossflow, this mode can also be carried out from a point source to a point drain (point-to-point). RED models show no large differences in generated power between co- and counter operation [8]. However, experimentally it was found that with co-flow a higher power was achieved [9]. This effect is attributed to the fact that in a stack with co-flow operation, the local pressure differences between adjacent flow channels are low in contrast to counterflow operation. In the last case there is more membrane deformation resulting in obstruction of the flow channels and more internal leakage.

Feasibility of RED is described by two response parameters: power density P_d_ (power per m^2^ total membrane) and energy efficiency η (the generated amount of electrical energy as part of the exergy from the inlet). In earlier experiments, we studied the effect of different parameters on these response parameters [8]. If was found that there is a tradeoff between power density and efficiency. As a compromise between both, a new response parameter was constructed: the response product, the product of efficiency and power density. However, in this paper, we restrict ourselves only to efficiency. Experimental power densities are typical 1–2 W/m^2^ [10]. If operated under conditions of maximal power density; the energy efficiency is about 20% [9].

In contrast to this rather low efficiency, Post et al. [11] showed an energy efficiency of above 85% with circulating feed waters (hydrodynamic losses were not considered). Because in real RED-applications flow through systems are generally used, this raises the question how to increase the efficiency of these systems to the values of Post et al. It turned out that the solution is a multistage system with an optimal load for each stage. Veerman et al. [2] performed experiments using feed water of 30.8 and 0.34 g NaCl/L in a RED stack, equipped with a total of one square meter ion exchange membranes. The load was adjusted for maximum power production which was achieved with a current of 300 mA. The outlet concentrations were measured and in a second experiment the same RED stack was operated with these outlet concentrations and again with 300 mA current. This was repeated a third and a fourth time, simulating a four stage setup. The harvested power of each of the four experiments was 0.83, 0.53, 0.26 and 0.03 W, a total of 1.65 W. Thus, the total power was doubled in the four stage experiment using four times more membrane area. The power density (in W/m^2^) was halved while the energy efficiency doubled indicating a tradeoff between power density and efficiency. Because the same amperage was applied in every experiment, it was demonstrated that with a real four-stage setup it is also possible to connect the four units electrically in series; in that case only a single converter is needed to supply the generated power to a local network.

Vermaas et al. [12] developed a process model for co- counter- and crossflow operation of a RED stack and focused on the energy efficiency of this system. Figure seven shows the main results for operation with equal flows for both feed waters. Comparing the three flow-options, it was found that almost the same energy efficiency was achieved with counter- and crossflow—much more than with co-flow. However, the internal losses in counterflow was about twice that of crossflow. Thus, the outlet of a crossflow operated stack has a relatively high exergy content and this flow mode is the best choice for closed circuit RED as applied at energy storage systems and heat-to-power generators.

Tedesco et al. [13] developed a process simulator and investigated the influence of various process variables, including a number of different configurations of multistaging. It was found that the best performance of the different arrangements was dependent on the relative availability of the high and low concentrated feed waters.

Hu et al. [14,15] investigated two strategies for maximum power extraction in a multistage RED device. In strategy I, each stack has its own load and in II, all stacks were electrically connected in series and conducted therefore the same current. The conclusion was that the difference in energy yield is small and strategy II is more feasible due to the simpler control and due to the lower costs because only one convertor is needed.

Another way to reach higher efficiencies is using segmented electrodes which divide the stack into a number of independent sections. The advantage is that segmentation can be applied in a single stack. Disadvantages are the possible mutual influences between the different sections. Therefore, each section must also be provided with a separate converter. This method also lacks the possibilities (in contrast to multistaging) to maximize the design of each stage individually with special membranes, spacers or residence times for the feed waters. In an experiment of Veerman et al. [9] an unsegmented RED stack delivered 8.23 W electrical power, whereas a total power of 9.10 W was achieved with three electrode segments, a gain of 11%. Simões et al. [16] applied segmentation to a crossflow stack. The flow directions in this RED stack—as developed by REDstack bv, The Netherlands—are mutually perpendicular as depicted in Figure 2c. By dividing the electrode in four segments, the power density was increased with 43% and the net energy efficiency with 39%.

As in RED, also in normal ED the profits of multistaging were seen ([17,18,19,20,21]). The guiding idea is the principle of equipartition of entropy production as described by Tondeur and Kvaalen [22]. Moreover, also in ED, electrode segmentation may be advantageous as an alternative to improve the efficiency and its application was investigated by Doornbusch et al. [23].

In practice three kinds of stack operation are used: co-flow, counterflow and crossflow. For each of them Vermaas et al. [12] developed and validated models. These are complicated models which are in good agreement with experimental results, but difficult to adapt to new requirements. In this study, simple models for co- and counter operation will be derived and applied to different multistage configurations.

In our simple model, we assume:

- Ideal NaCl solutions;
- Concentrations of 1 and 30 kg/m^3^;
- A temperature of 25 °C;
- Stacks with only one cell pair;
- Feed-water-flow rates of 1 m^3^/s. (If divided by 10^6^, flow rates are in the range of normal lab stacks).

## 2. Efficiency of the RED process

### 2.1. Energy Efficiency and Thermodynamic Efficiency

If concentrations are not too high, the flow rate of Gibbs energy *X*—known as *exergy flow*—of a certain flow rate river water together with seawater can be derived using the equation of Forgacs [24]:

(1) $$X=Cr\Phi r\ln\frac{Cr}{Cm\;}+Cs\Phi s\ln\frac{Cs}{Cm\;}\;\mathrm{with}\;Cm=\frac{Cr\Phi r+Cs\Phi s}{\Phi r+\Phi s}$$

where *R* stands for the gas constant, *T* the temperature, *Cr* and *Cs* the concentrations of river and seawater, Φ*r* and Φ*s* the flow rates of river and seawater and *Cm* the concentration of the mixture of both solutions.

There are two different ways to define the efficiency of a RED stack. In a flow through system usually the *energy efficiency* (*η*) is used. This value can be related to the pure electrical power (*η_gross_*) or to the net power as corrected for hydrodynamic losses (*η_net_*):

(2) $$\eta_{gross}=P/X_{in}$$

(3) $$\eta_{net}=\left(P-P_{hydr}\right)/X_{in}$$

where *P* stands for the generated electrical power and *P_hyd_*_r_ for the hydrodynamic loss. In closed systems as applied in energy storage and heat-to-power systems, the salinity gradient in the outlets can be used again. Here sometimes the thermodynamic efficiency (ε) is used:

(4) $$\varepsilon_{gross}=P/\left(X_{in}-X_{out}\right)$$

This parameter can also be corrected for hydrodynamic loss:

(5) $$\varepsilon_{net}=\left(P-P_{hydr}\right)/\left(X_{in}-X_{out}\right)$$

As stated earlier, Post et al. [11] achieved with a closed system gross energy efficiencies until 85%, dependent on the applied electrical current. This can be explained as follows: In an ideal RED system the power is dissipated inside the stack and into an external resistance. The power efficiency *ε_power_* is the fraction of this power in the external resistance:

(6) $$\varepsilon_{power}=i\cdot R_{e}/\left(i\cdot R_{e}+i\cdot R_{i}\right)=R_{e}/\left(R_{e}+R_{i}\right)$$

where *i* stands for the electrical current and *Re* and *Ri* for the external and internal resistance. In an ideal system *ε_power_* is equal to *ε_gross_* as defined in Equation (4). The internal resistance in a closed system is dependent on time and in a flow through system dependent on the position in the flow path. However, a high thermodynamic efficiency is achieved by a low power output (a high *Re*) and the feed water volume must be recirculated many times to reach a large scale of energy yield. This implies a high input of pump energy and the net effect of a high gross thermodynamic efficiency in a closed circuit system results therefor in a low (or negative) net thermodynamic efficiency.

For maximum thermodynamic efficiency, *Re* should be much larger than *Ri*. In that case, a theoretical conversion near 100% is possible. The power *P* of the device is:

(7) $$P=i^{2}\times Re=\;\;{\left(\frac{E}{Ri+Re}\right)}^{2}\;Re$$

where *E* stands for the EMF (electromotive force) and *i* for the electrical current. If we assume a fixed *Ri* and a variable *Re*, we can find the maximum power by differentiating *P* to *Re* and making the result equal to 0:

(8) $$\frac{dP}{dRe}=\;\;E^{2}\frac{Ri-Re}{{\left(Ri+Re\right)}^{3}}=0$$

It follows that maximum power is achieved with *Re = Ri*. It should be emphasized in all above considerations that *Ri* is not constant; it is dependent on the concentrations in the feed water compartments and decreases during the process in the closed circuit system.

### 2.2. Co-flow Operation

**Figure 3:**
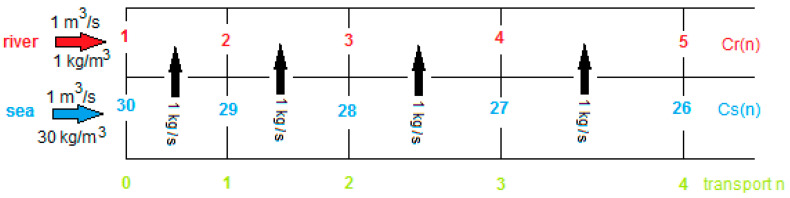
Entrance of a RED stack operated in co-flow. Red numbers indicate the salt concentration Cr(n) in the river water compartment and blue the concentration Cs(n) in the seawater compartment. Green numbers indicate the transport parameter n.

In the stack there is a salt transport from the seawater compartment (S) to the river water compartment (R). The concentration in R increases following the flow path while the concentration within S decreases. The electromotive force *E* can be calculated as function of the transport parameter *n*:

(9) $$E\left(n\right)=2\frac{RT}{zF}\ln\left(\frac{Csi-n}{Cri+n}\right)$$

with *n* the transported amount of salt (kg/s), R the gas constant, T the temperature, *F* the constant of van Faraday, *z* the charge of the concerning ion (in this case *z* = 1 for Na^+^ as well as for Cl^−^) and *Csi* and *Cri* the inlet concentrations of *S* and *R* (kg/m^3^). The factor 2 is due to the combination of a CEM with an EM to a cell pair. Figure 3 shows the entrance of the stack and the salt balance. Due to the decreasing driving force from inlet to outlet, the distances in which 1 kg salt per second is transported will increase. A schematic representation of this phenomenon with EMF of the stack as function of the salt transport *n* is shown in this figure.

In Figure 3 it is seen that the sum of the concentrations in *S* and *R* are independent of the location in the RED stack.

(10) $$Cr\left(n\right)=Cri+n\;\;\;;\;\;\;Cs\left(n\right)=Csi-n\;\;;\;\;Cr\left(n\right)+Cs\left(n\right)=Cri+Csi$$

Here, *Cri* and *Csi* are the inlet concentrations in kg/m^3^.

**Figure 4:**
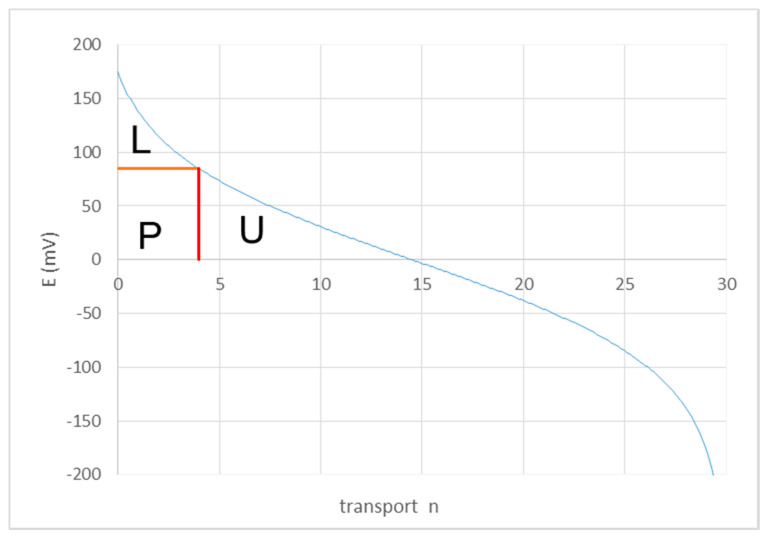
Cell EMF as function of the salt transport *n*. The behavior or a normal RED stack operated at maximum energy efficiency is described by the part of the graph left from *n* = 4.

Figure 4 shows the EMF *E* as function of the transport parameter *n* according to Equation (9). In RED applications, only a part of the sketched EMF curvature applies. Only with a strong negative applied potential, the complete curvature can be constructed; this RED method is known as ARED (assisted reverse electrodialysis) [25]. In normal RED operation the stack is connected with an Ohmic load, resulting in a clamp voltage *Uc* which is lower than the EMF *E*. In Figure 4 the clamp voltage is 85 mV, corresponding to a transport of about 4 kg/s. As stated earlier, the sections of equal transport in the stack increase in the direction of the flow and the final part (with *n* from 4 to 5 kg/s) is even infinitely long. Therefore, the outlet R concentration will be a little below 5 kg/m^3^.

The electrical current *i* is proportional to the salt transport *n*:

(11) $$i=\frac{n}{M}F$$

with *n* the salt transport (kg/s), *M* the molecular mass of NaCl (kg/mol) and *F* de Faraday constant (C/mol).

In Figure 4 the harvested power is indicated by the rectangle P, the Ohmic loss by L and the unused part of the exergy that leaves the device again by U (‘unused’). With given flow rates of *R* and *S*, the maximum delivered power *P* can be achieved in practice by adjusting the load. In this case the rectangle *P* is also at its maximum:

(12) $$P\left(n\right)=\frac{n}{M}F\times E\left(n\right)=\frac{n}{M}F\times 2\frac{RT}{zF}\ln\left(\frac{Csi-n}{Cri+n}\right)=n\frac{2RT}{M}\ln\left(\frac{Csi-n}{Cri+n}\right)$$

Here, *Cri* and *Csi* are inlet concentrations. By maximizing *P*, the optimal *n* (*n_opt_*) can be easily found; with inlet concentrations of 1 and 30 kg/m^3^, the value of *n_opt_* is 6.495. The corresponding clamp voltage *Uc* is 59 mV and the delivered power *P* is 630 kW. Percentages of the different values are compared with data from Vermaas et al. [12] in Figure 7. The values of our simple model—in fact only Equation (12)—differ only marginally from the sophisticated model of Vermaas et al. differences.

An alternative parameter for the progress of the process is the mixing degree, *MD*:

(13) $$\mathrm{MD}=\frac{Cro-Cri}{Cm-Cri}$$

**Figure 5:**
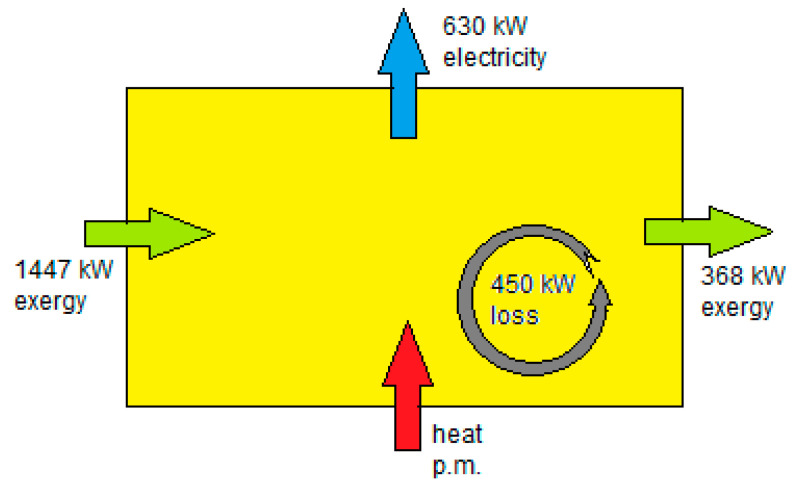
Relation between heat, energy and exergy flows in a RED stack with co-flow feed. Green arrows indicate exergy flows via the feed waters. Normal stack operation is adiabatic, and the consequence is that the outgoing feed waters are a little cooler than the incoming feed waters.

Here, *Cm* is the concentration obtained with total mixing as mentioned in Equation (1). In this case it turns out that MD = 44.8%. With the equation of Forgacs (Equation (1)) it is possible to calculate the in- and outgoing exergy stream. The result is X_in_ = 1447 kW and X_out_ = 368 kW. From this follows the Ohmic loss (Loss = Ex_in_ − Ex_out_ − P), resulting in Loss = 450 kW. These values are depicted in Figure 5.

In the above consideration, the maximum power was calculated at fixed flow rates of 1 m^3^/s for both *R* and *S*. Maximum power therefore means maximum use of the exergy of the incoming feed water. Therefore, output concentrations are linked to this most efficient setting. Control of these starting concentrations can only be done with an existing stack by adjusting the residence time.

### 2.3. Counterflow Operation

**Figure 6:**
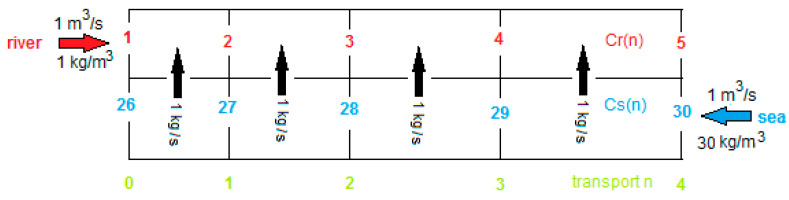
A RED stack with counterflow operation. Red numbers indicate salt concentrations in the river water compartment, blue in the seawater compartment. Transport parameter is given in green.

Figure 6 shows a counterflow RED stack with input concentrations of 1 and 30 kg/m^3^. The system is defined by the output concentration of the river water flow; in the example of Figure 6 this is 5 kg/m^3^. In contrast to the co-flow mode, where there is an equal sum of concentrations at every location in the stack, there is now a constant difference:

(14) $$Cs\left(n\right)-Cr\left(n\right)=Csi-Cro$$

with *Cs(n)* and *Cr(n)* the location dependent concentrations in the sea and river water compartments, *Csi* the sea water inlet concentration and *Cro* the river water outlet concentration.

The delivered power of a stack is the product of electrical current and the lowest EMF in the range between inlet and outlet. This lowest EMF is situated at the river water outlet and the optimal transport parameter at this place is *n* = *n_opt_*. The EMK of the stack is:

(15) $$E\left(n_{opt}\right)=\frac{RT}{zF}\ln\left(\frac{Csi}{Cro}\right)=\frac{RT}{zF}\ln\left(\frac{Csi}{Cri+n_{opt}}\right)$$

If *n* = *n_opt_*, the produced power is maximum and is the product of Equation (11) and Equation (15):

(16) $$P\left(n_{opt}\right)=\frac{n_{opt}}{M}F\times \frac{RT}{zF}\ln\left(\frac{Csi}{Cri+n_{opt}}\right)=\frac{n_{opt}}{M}RT\ln\left(\frac{Csi}{Cri+n_{opt}}\right)\;\;\;\;with\;z=1$$

Therefore, the following function should be maximized by adjustment of *n_opt_*:

(17) $$P\left(n_{opt}\right)\;∼n_{opt}\cdot \ln\left(\frac{Csi}{Cri+n_{opt}}\right)$$

**Figure 7:**
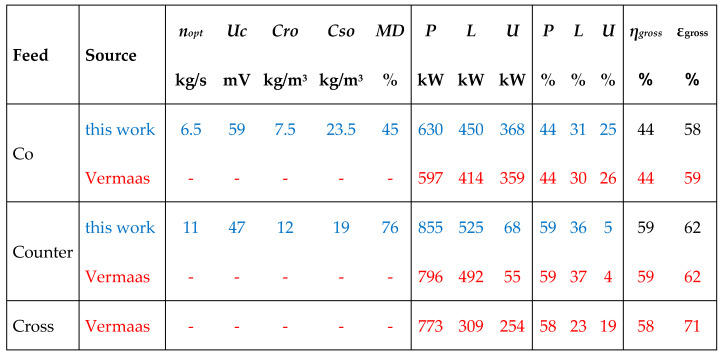
Results of this work for stacks with inlet concentrations 1 and 30 kg/m3 compared with data of Vermaas et al. [12]. *n_opt_* stands for the optimal transport, *U_c_* for the clamp voltage, *Cro* and *Cso* for the river and seawater outlet concentrations, *P* for generated electrical power, *L* for loss and *U* for unused exergy; *η_gross_* and *ε_gross_* are the energy efficiency and the thermodynamic efficiency.

In this case too, the maximum power of *P* is easy to find using the Excel solver. Results (with input concentrations 1 and 30 kg/m^3^ at 25 °C) of co- and counterflow are shown in Figure 7 and compared with data from Vermaas et al. [12]. Again, there is a strong resemblance of our results using only Equation (17) and the results of the complex model of Vermaas et al. [12]. The thermodynamic efficiency is important for closed systems; the unused part will be used again in subsequent cycles. This is in contrast to flow-through systems such as Blue Energy where the energy efficiency plays an important role.

## 3. Multistaging

**Figure 8:**
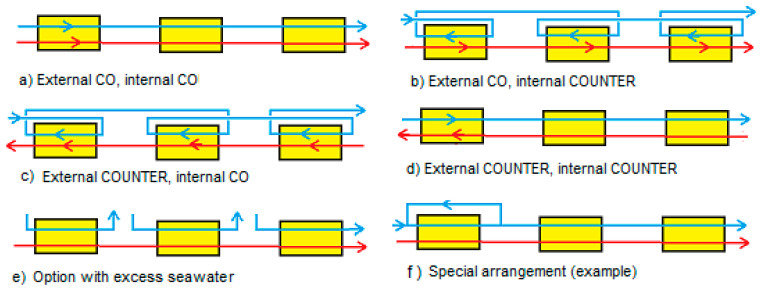
Different multistage systems. Systems externally operated in (**a**,**b**) co-flow mode; (**c**,**d**) counterflow mode. Internal co-flow is seen in (**a**,**c**), whereas internal counterflow occurs in (**b**,**d**). Systems (**a**–**d**) are the subject of this article. System (**e**) is an option if there is a large excess of seawater (blue flow paths) available. System (**f**) is an example of numerous other interconnections and is taken from Tedesco et al. [13].

Multistaging is connecting different stacks with the aim of achieving a higher efficiency or power density. We restricted us to co- and counter fed stacks which were interconnected also in co- or counterflow as shown in Figure 8. Here (a) and (b) represent co-flow interconnection whereas (c) and (d) stand for counterflow interconnection. Internal co-flow is shown in (a) and (c) while internal counterflow is depicted in (b) and (d). In our approach al modification are fed with 1:1 l flow rates of feed waters. There are numerous possibilities for other stack arrangements: (e) is an example of operation with an excess of seawater (blue flow paths); from Tedesco et al. [13] is taken (f) as an example of one of the arrangements they use.

For optimization of the energy efficiency, we applied three different methods:

- Method A. The power of the first stack is maximized by adjusting the transport parameter *n_1_*. Then, this process is repeated with the second stack with *n_2_* and so on.
- Method B. The total delivered power is maximized by simultaneous adjustment of the individual transport parameters *n_1_*, *n_2_*, *n_3_*...
- Method C. All transport parameters are made equal to each other: *n* = *n*_1_ = *n*_2_ = *n*_3_... and then the total power is maximized by adjusting this *n*.

Method A, B and C are applied to arrangements (a) and (b) while method B and C are applied to (c) and (d).

**Figure 9:**
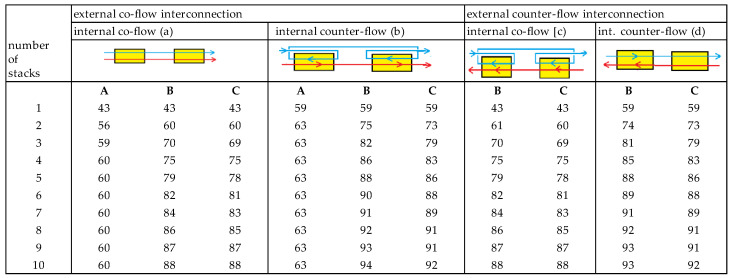
Energy efficiency in percent for the different arrangements of co-flow and counterflow stacks. Three methods for optimization the energy efficiency are listed: with method A successively the power of each stage is maximized, with B the power of the whole system is maximized by adjusting the electrical current in all stages are simultaneously, and with C the power of the whole system is maximized by adjusting the same current through each stage.

All calculated efficiencies are listed in Figure 9. The highest energy efficiencies appear in arrangement (b), (co-flow of stacks with internal counterflow). Moreover, within arrangement (b), optimization method B result in a little higher values than method C. However, the advantage of method B (each stack the same electrical current) is that all stacks can be connected in series, and only one convertor is needed for delivering the power to the local network.

**Figure 10:**
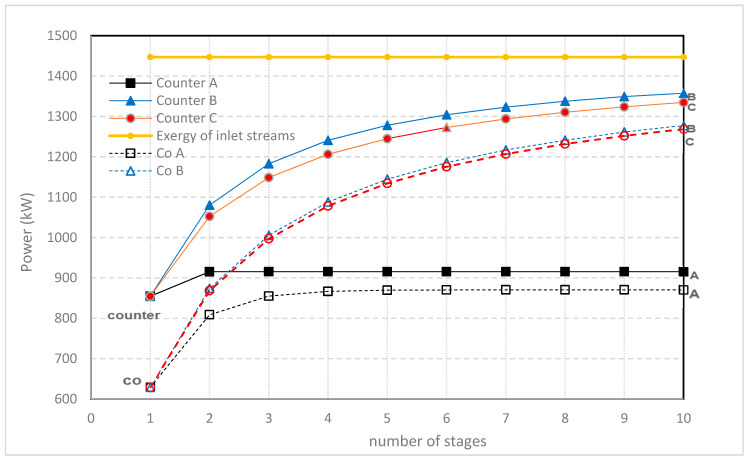
Power in kW with multistaging in co-flow stacks (dashed lines; added to aid interpretation) and counterflow stacks (solid lines). Stacks are external connected in co-flow. Feed-water-flow rate is 1 m^3^/s, concentrations 1 and 30 kg/m^3^. Three different optimizing methods (A, B and C) are used (see caption Figure 9).

Results of co- and counterflow with co-flow between the stack are also plotted in Figure 10. Here, the produced electrical power is plotted against the number of stages. Differences between co- and counterflow decrease if more stages are applied. It is known that counterflow can introduce some problems due to the pressure gradient between *R* and *S* compartments (internal leakage and flow path deformation) and therefore the combination of internal co-flow with external co-flow seems a good option together with electrical serial interconnection.

## 4. Conclusions

By using multistaging, the energy efficiency of the RED process can be improved significantly; in the case of internal co-flow even by a factor of 2. Adding extra stages reduces the average power density, but on the other hand, the available feed water is used more economical. The usefulness of this is evident in the case of feed water scarcity, but energy efficiency is also important because less feed water is needed per kWh produced energy and therefore less energy is lost for the necessary pretreatment.

If only a single stack is used, then internal counterflow outperforms co-flow significantly. However, as the number of stages increases, the difference narrows. In practice, internal co-flow has the advantage that the local pressure differences between *S* and *R* are minimal. Then there is less internal leak and less distortion of the flow channels. The latter leads to an increased pressure drop and a less uniform flow through the compartments, resulting in higher hydrodynamic losses and hindered ion transport through the membranes.

Using external counterflow has no advantages while the possible problems that can arise from the pressure differences are even exaggerated. Moreover, in practice the technique is more complicated.

Optimization methods B and C perform significantly better than method A. There are no major differences in efficiency between B and C. Method B, however, requires a separate electronic converter for each stage. In method C, there is an equal amperage in the various stages so they can connect in series and only one convertor is needed for the multistage system. This is a cheap solution that is also easy to control because only one parameter needs to be optimized—exactly the same as that of a separate stack. A requirement is then that the stages are reasonably isolated from each other, which can be achieved with relatively long connecting tubing between the stages.

Modeling the optimization process is very simple because the behavior of a stage is described by only one equation: Equation (12) for co-flow and Equation (17) for counterflow operation. This makes it possible to maximize the energy efficiency of complex multistage systems with simple standard software.

If we compare multistaging with electrode segmentation, several things stand out. As stated before, with multistaging it is possible to harvest the energy by electrical connecting all stacks in series. Obviously, this is not possible with a segmented electrode system where the different segments are in lateral contact. Multistaging also makes it possible to take in account the changing salinity gradient in the different stages. Thicknesses of the compartments and type of membranes can be adjusted to local conditions. On the other hand, electrode segmentation multistaging can be applied within a certain stack design and can have a positive influence on both energy efficiency and power density. The question remains whether electrode segmentation still makes sense if the stack gets many cell pairs. The lateral influence of the various segments will no longer be negligible in stacks with many cell pairs.

## Nomenclature

*Roman*	
C	concentration (mol/L)
E	electromotive force (V)
F	Faraday constant (96 485 C∙mol^−1^)
i	electrical current (A)
Loss	internal loss in the stack (W)
M	molecular weight of NaCl (0.05844 kg/mol)
MD	mixing degree
n	transport parameter (kg/s)
R	gas constant (8.32432 J∙mol^−1^ K^−1^)
Re	external resistance of a RED stack (load) (Ω)
Ri	internal resistance of a RED stack (load) (Ω)
P	power (W)
T	temperature (K)
Uc	clamp voltage (V)
V	volume (m^3^)
X	exergy flow (W)
*Greek*	
*ε*	thermodynamic efficiency
*η*	energy efficiency
Φ	flow rate (m^3^/s)
*Subscripts*	
hydro	hydrodynamic
i	inlet
o	outlet
opt	optimal
r	river
s	sea
*Acronyms*	
EM	anion exchange membrane
CEM	cation exchange membrane
ED	electrodialysis
EMF	electromotive force
RED	reverse electrodialysis
